# Effect of Co-contamination by PAHs and Heavy Metals on Bacterial Communities of Diesel Contaminated Soils of South Shetland Islands, Antarctica

**DOI:** 10.3390/microorganisms8111749

**Published:** 2020-11-07

**Authors:** Alejandro Gran-Scheuch, Javiera Ramos-Zuñiga, Edwar Fuentes, Denisse Bravo, José M. Pérez-Donoso

**Affiliations:** 1 BioNanotechnology and Microbiology Lab, Center for Bioinformatics and Integrative Biology (CBIB), Facultad de Ciencias de la Vida, Universidad Andres Bello, Republica # 330, Santiago 8370146, Chile; agranscheuch@gmail.com (A.G.-S.); ramoszjaviera@gmail.com (J.R.-Z.); 2 Departamento de Química Inorgánica y Analítica, Facultad de Ciencias Químicas y Farmacéuticas, Universidad de Chile, Sergio Livingstone Pohlhammer # 1007, Santiago 8380000, Chile; edfuentes@ciq.uchile.cl; 3 Laboratorio de Microbiología Oral, Facultad de Odontología, Universidad de Chile, Sergio Livingstone Pohlhammer # 943, Santiago 8380453, Chile; denisseb@gmail.com

**Keywords:** Antarctica, co-contamination, microbial population, PAHs, cadmium, PAH-degrading bacteria

## Abstract

Diesel oil is the main source of energy used in Antarctica. Since diesel is composed of toxic compounds such as polycyclic aromatic hydrocarbons (PAHs) and heavy metals, it represents a constant threat to the organisms inhabiting this continent. In the present study, we characterized the chemical and biological parameters of diesel-exposed soils obtained from King George Island in Antarctica. Contaminated soils present PAH concentrations 1000 times higher than non-exposed soils. Some contaminated soil samples also exhibited high concentrations of cadmium and lead. A 16S metagenome analysis revealed the effect of co-contamination on bacterial communities. An increase in the relative abundance of bacteria known as PAH degraders or metal resistant was determined in co-contaminated soils. Accordingly, the soil containing higher amounts of PAHs exhibited increased dehydrogenase activity than control soils, suggesting that the microorganisms present can metabolize diesel. The inhibitory effect on soil metabolism produced by cadmium was lower in diesel-contaminated soils. Moreover, diesel-contaminated soils contain higher amounts of cultivable heterotrophic, cadmium-tolerant, and PAH-degrading bacteria than control soils. Obtained results indicate that diesel contamination at King George island has affected microbial communities, favoring the presence of microorganisms capable of utilizing PAHs as a carbon source, even in the presence of heavy metals.

## 1. Introduction

Antarctica has many natural resources and contains near 80% of the freshwater reserves of our planet. Because of its strategic relevance and unique characteristics, over 30 nations have established scientific and military facilities on this continent, including the United States, China, Germany, Russia, Chile, and the United Kingdom. Moreover, the tourism of the continent has increased during the last decade. As a consequence of human exploration and the growing tourism industry, anthropogenic pollution of Antarctica has become a concern in the last few decades [1,2,3,4].

Historically, most human activities on the continent have involved the use of diesel-based fuels for transport and energy. In this context, diesel contamination represents one of the oldest and most significant environmental problems of the continent [4]. This situation is particularly relevant for scientific stations where oil consumption usually exceeds 100,000 L per year, and accidental spills occur due to the storage and refueling of vehicles and aircraft [2,5]. Moreover, deterioration of storage tanks through time has contributed to the release of diesel and other pollutants, increasing the environmental damage.

Diesel oil is composed of toxic compounds such as heavy metals and PAHs and constitutes a constant threat to organisms exposed to it. In general, PAHs’ toxicity depends on their chemical structure; however, they share some characteristics like being mutagenic, teratogenic, carcinogenic, and highly recalcitrant to degradation [6,7,8]. The toxicity of heavy metals is mostly associated with the generation of oxidative stress that damage cellular membranes, proteins, enzymes, and DNA [9,10].

During the last few decades, many studies have quantified the levels and effects of diesel contamination in Antarctica [2,4,8,11,12,13,14]. Most Antarctic studies have focused on the Ross sea region, with a minority percentage in the Shetland Islands, the Antarctic Peninsula, and East Antarctica [2]. In this context, high levels of PAHs and heavy metals ] determined in diesel contaminated soils of different regions of Antarctica. The quantification of diesel-derived toxic elements in Antarctic and sub-Antarctic areas revealed the existence of several contamination hotspots near human activity zones [2,15,16,17,18]. The presence of different PAHs like naphthalene, phenanthrene, and fluorene has been detected in various studies, mainly associated with diesel contamination [2,11,14]. Additionally, increased concentrations of lead, cadmium, chromium, zinc, and nickel have been determined in different zones of Antarctica [2,15,19,20,21]. In particular, high levels of lead and cadmium have been determined at the proximity of fuel tanks near scientific bases [15,20,22].

Co-contamination of PAHs and metals is a topic scarcely studied to date; however, it is known that the mixture of these compounds damage all kinds of organisms and strongly affects bioremediation processes [23,24]. Recent reports revealed the effect of metals over different metabolic enzymes involved in PAH biodegradation [25,26,27]. In particular, heavy metals like cadmium inhibit oxygenase enzymes that directly participate in PAHs’ metabolism [24,28].

In this context, the presence of PAHs and heavy metals on plants, birds, algae, and microorganisms has been documented [29,30,31,32,33,34,35]. In the case of microorganisms, an increase in the number of hydrocarbon-degrading bacteria has been reported in different studies [36,37,38,39]. However, microorganisms’ real contribution to diesel degradation in Antarctic soils has not been deciphered, and their potential for in situ bioremediation strategies has not been fully determined. It has been suggested that diesel remediation of Antarctic soils is mostly a consequence of volatilization, with a minor contribution of microorganisms to the process [40]. In this regard, just a few works have attempted to determine the impacts of volatilization and biodegradation on the rate of PAH-degradation in Antarctic contaminated soils [12]. On the other hand, more recent studies indicate that the metabolic activity of PAH-degrading bacteria is a significant component of diesel degradation in Antarctic soils and have proposed the use of these microorganisms for in situ bioremediation strategies [2,36,37,38].

Several factors determine the final effect of diesel contamination on the microbial composition of soils. Hydrocarbon content, but also the presence of other elements, determines the final effect of diesel on the dynamics of soil microbial communities [39]. Interestingly, Aislabie et al. in 2001 reported no changes in bacterial hydrocarbon degraders in diesel-contaminated soil at the Scott base, a result that was associated with the high concentrations of lead in soils [41]. Other works have described the effects of diesel and other hydrocarbons on microbial communities inhabiting marine sediments and nearshore environments in Antarctica [42,43]. To date, the importance of PAHs and heavy metal co-contamination on diesel biodegradation or in the dynamics and properties of soil microbial communities is still unknown. However, there is increasing evidence suggesting that co-contamination affects the metabolic capabilities of bacterial cells.

In previous work, we studied diesel contaminated soil samples and isolated microorganisms capable of efficiently degrading phenanthrene (a PAH present in diesel) [44]. Interestingly, the isolate exhibiting the highest capacity to degrade this PAH, *Sphingobium xenophagum* D43FB, was capable of doing it even in the presence of cadmium. These results suggest that the presence of these compounds in diesel contaminated Antarctic soils are probably selecting for microorganisms capable of utilizing PAHs in the presence of heavy metals.

In the present work, we studied the presence of harmful diesel components such as PAHs and heavy metals in soils of King George Island, Antarctica. We also determined the effect of co-contamination on dehydrogenase activities of bacterial soil communities and how this contamination affects the number of culturable bacteria.

## 2. Materials and Methods

### 2.1. Reagents

Acetonitrile, hexane, acetone, methanol, and phenanthrene were obtained from Merck (Darmstadt, Germany). 2,3,5-Triphenyl tetrazolium chloride (TTC) and cadmium chloride were obtained from Sigma-Aldrich (analytical grade; San Luis, MO, USA). Bacterial growth media ingredients were purchased from BD Difco (Sparks, MD, USA). Reasoner 2A (R2A) and minimal growth media M9 were used for bacterial growth [45]. M9 was supplemented with anhydrous glucose or phenanthrene at 0.2% *w*/*v*.

### 2.2. Soil Sampling

A total of 24 soil samples from 8 different sites were used in this study (the number of soil samples analyzed was determined by Antarctic Chilean Institute regulations and is the same used in similar studies of this island) [39]. The soils selected were evenly distributed across different geographic sites across King George Island in Antarctica (62°10 S, 58°49 W), see map station (Figure S1). Sampling was conducted during February 2013, where the environmental conditions in sampling spots were similar. Soil samples were collected from surface soil (0–10 cm) at four sites with no visible diesel contamination (designated A, B, C, and D), and four sites exposed to diesel located near the Chilean research facility (E, F, G, and H) (Table 1). For the selection of the sampling sites, places that did not show evident human interference, nor the presence of surface vegetation, mosses, or lichens were chosen. Surface soil samples (approximately 500 g) were obtained, sealed in sterile sampling tubes, and transported to the laboratory on ice.

### 2.3. Analysis of Chemical Parameters

The pH of soil samples was determined in triplicate by suspending 1 g of soil in 10 mL of distilled water, then the suspension was vortexed and after 5 min the pH was measured with a glass electrode [46]. Quantitative analysis of the three-ring PAHs family was performed by total fluorescence spectroscopy with multivariate data analysis as described previously [47]. Calibration was performed by measuring the excitation–emission matrices of standard solutions of phenanthrene at different concentrations in hexane. Benzo[a]pyrene was used in orthogonal concentrations with respect to phenanthrene as possible interference in the matrix. The validation was performed by analyzing soil samples amended with known concentrations of phenanthrene and benzo[a]pyrene through multivariate calibration. Excitation–emission matrices were analyzed by MATLAB software (v.7.6, Mathworks, Natick, MA, USA) to predict the concentrations of phenanthrene in soil extracts. PARAFAC and U-PLS/RBL methods were used. The method has a prediction error of 10% [48]. The extraction of nonpolar compounds from 1 g of soil samples was made in duplicate by adding 5 mL of hexane and 1 mL of acetonitrile: water (70:30). The extractions were assisted by microwave; with a program of 250 W for 2 min, 750 W for 10 min, and then cooled for 10 min. A 5 mL final volume was obtained by adding hexane [49,50]. Finally, total fluorescence spectra (emission from 220 to 400 nm every 5 nm, and excitation from 324 to 550 nm every 2 nm) were determined. Total concentrations of cadmium, lead and chromium in soil samples were quantified by ICP-MS using the commercial service of Comisión Chilena de Energía Nuclear (CChen), Chile (Table S1).

### 2.4. Analysis of Biological Parameters

Soil dehydrogenase activities were determined using a previously described method with some modifications [51]. An amount of 560 µL of TTC 0.85% *w*/*v* prepared in sterile distilled water (or in a sterile cadmium solution at 12 or 25 mg kg^−1^ final concentration for the Cd tolerance test) was added to 1 g of sample. Solutions were prepared in amber vials and incubated one week at 8, 18, or 28 °C. Then, 1600 µL of methanol was added twice to extract the insoluble red dye triphenyl formazan (TPF) generated from TTC reduction. Mixtures were centrifuged at 10,000 r.p.m. for 5 min, supernatants recovered and the absorbance at 485 nm determined. The quantification was performed using a calibration curve constructed using TPF solutions prepared in methanol. Colony-forming units per gram of soil (CFU g^−1^) were determined by a previously reported procedure with some modifications [52]. Briefly, 1 g of soil sample was suspended in 1 mL of sterile distilled water, stirred for 1 h at 20 °C, and serially diluted in 96-wells microplates, in triplicate. Then, 10 μL of each dilution was spotted on R2A solid media (in presence or absence of cadmium 12 or 25 mg L^−1^) and incubated at 8, 18, or 28 °C for 48 h.

### 2.5. 16S rRNA Gene Analysis

Three soil samples were used for 16S rRNA gene analysis: A (non-exposed), E, and F (diesel-exposed zones). DNA extraction was performed by using PowerSoil^®^ DNA Isolation Kit from Qiagen (Hilden, Germany) and then total DNA was quantified using Qubit fluorometer (Invitrogen, Carlsbad, CA, USA). Samples were sequenced in the Argonne National Laboratories using the Earth Microbiome Project barcoded primer set, adapted for the Illumina HiSeq2000 and MiSeq [53,54]. Data analysis of the 16S rRNA gene for microbial communities was performed using the DADA2 software package for Illumina sequencing paired-end fastq files (https://benjjneb.github.io/dada2/) [55]. Alpha diversity and abundance analysis were carried out using the phyloseq R package (https://joey711.github.io/phyloseq/) [56].

### 2.6. Isolation of Phenanthrene Degrading Bacteria

Phenanthrene-degrading bacteria isolation was performed using a previously described method [44]. A three-step enrichment and screening process was followed. Soil samples were suspended in sterile distilled water (50% *w*/*v*), supplemented with UV-light sterilized phenanthrene (20% *w*/*v*), and incubated 72 h at 8, 18, and 28 °C. Subsequently, supernatants were plated in Reasoner’s 2 (R2A) agar and incubated at 8, 18, or 28 °C until colony growth was observed. Colonies with different morphologies were isolated. Then, each isolate was inoculated in R2A culture media. After 24 h, suspensions were centrifuged at 7000 r.p.m. for 5 min and the pellet was washed twice with a sterile 25 mM phosphate buffer. Finally, obtained solutions were used to inoculate (1:1000) M9 minimal media supplemented with phenanthrene (2000 mg L^−1^) as the only carbon source. Cultures were grown with agitation at room temperature for 5 days, and the growth of phenanthrene-degrading bacteria was screened by culture turbidity and change of color in the culture due to the generation of 2′-hydroxy muconic semialdehyde, a degradation product of phenanthrene [57].

### 2.7. Data Analysis

Statistical analyses were performed using GraphPad Prism v5 for Windows (GraphPad Software, La Jolla, CA, USA). One-way ANOVA with a Tukey multi comparison test was used to establish statistical differences between the groups. The mean of each data set was compared between them using a *p* < 0.05. The Principal Component Analysis (PCA) was performed considering all parameters determined in the study (PAHs, metals, and biological activity) and using the “The Unscrambler” software (version 9.7, CAMO PROCESS AS, Oslo, Norway) as described before [58].

Algorithmic prediction for the determination of PAHs was performed using MATLAB (MathWorks Inc 2008) program with the algorithms “unfolded partial least squares with residual bilinearization” (U-PLS/RBL) and “parallel factor analysis” (PARAFAC). Both algorithms were implemented using the graphical interface MVC2. Routines to perform analyses are available on the internet http://www.models.kvl.dk/algorithms as is the graphical interface used MVC2 (http://www.chemometry.com).

## 3. Results

### 3.1. Antarctic Diesel-Exposed Soils Contain High Concentrations of PAHs and Heavy Metals

Chemical properties of selected soils such as pH, PAH concentration, and metal concentration were determined to characterize the levels of contaminants and their effects. Results indicate that soil samples exposed to diesel and controls have an average pH of 6.4 ± 0.35 and 6.8 ± 0.25, respectively (Figure S2). Tukey test data analysis suggests a significant difference between both groups, a situation that could be a consequence of different variables such as minerals, metals, contamination and/or biodiversity. Samples C and F display pH values differing from the average with pH values of 7.14 and 5.92 respectively; however, the differences are not statistically significant. Similar values have been reported in diesel-contaminated soils of Macquarie Island [39] and studies in other zones of the continent reported a slight decrease in the pH of diesel-contaminated soils probably as a consequence of the generation of organic acids derived from bacterial metabolism [5].

**Figure 1:**
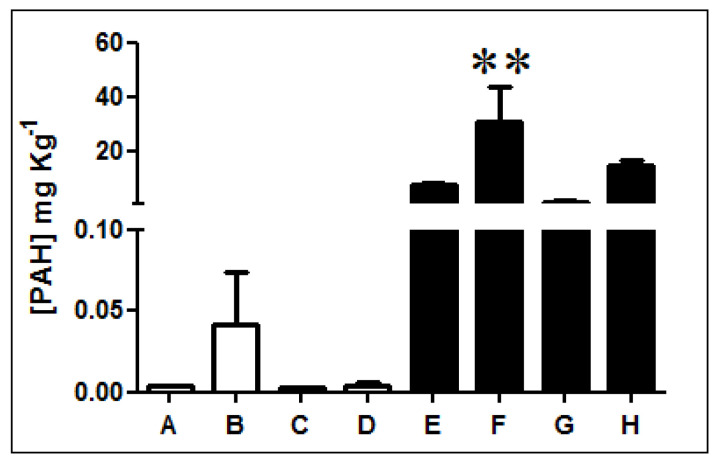
Quantification of three-ring PAHs in Antarctic soil samples. The concentration of three-rings PAHs was determined in non-exposed (white bars; A, B, C, and D) and diesel-exposed soil samples (black bars; E, F, G, and H). ** Represents significance with a *p* < 0.05.

Fluorescence analysis of three-ring PAHs revealed that diesel-exposed soil samples E, F, G, and H present higher concentrations of PAHs than non-exposed soils A, B, C, and D (Figure 1, Table S1). PAH quantification indicates that samples exposed to diesel present, on average, a PAH concentration 1000 times higher than non-exposed soils. In particular, sample F has the highest concentration presenting levels of PAHs over 2000 times greater.

**Figure 2:**
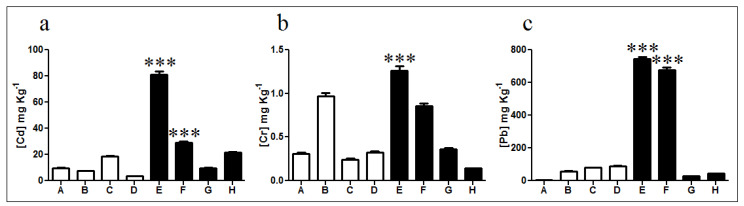
Quantification of heavy metals in soils. Concentration of (**a**) cadmium, (**b**) chromium, and (**c**) lead in non-exposed (white bars; A, B, C, and D) and diesel-exposed soil samples (black bars; E, F, G, and H). *** Represents significance with a *p* < 0.001.

Cadmium levels showed no significant differences in non-exposed soils, except for sample C which displayed almost twice the cadmium concentration of samples A, B, and D (Figure 2a). Furthermore, exposed sample E exhibited high levels of cadmium with a total concentration of 83 mg kg^−1^ corresponding to nearly 8 times the average cadmium content in non-exposed samples and 4 times the average concentration in exposed samples. Lead quantification showed similar values in all non-exposed soils with an average concentration of 58.2 mg kg^−1^ (Figure 2c). In diesel-exposed samples E and F lead concentrations of 743 and 678 mg kg^−1^ were measured, respectively. These concentrations are significantly different from those determined in samples G and H which presented only 29 and 44 mg kg^−1^, respectively. Finally, chromium quantification indicated a low presence in non-exposed soils, excepting sample B (0.97 mg kg^−1^) with almost three times the average concentration determined for non-exposed samples (Figure 2b). Diesel-exposed samples E and F presented 1.26 and 0.84 mg kg^−1^ of chromium, respectively. These concentrations represent near 3 and 7 times the levels determined on samples G and H, respectively.

Metal determinations indicated a significant increase in cadmium, lead, and chromium concentration particularly for diesel contaminated soils E and F, which agrees with the PAH concentrations determined in Figure 1.

### 3.2. Bacterial Communities of Diesel-Exposed Soils

**Figure 3:**
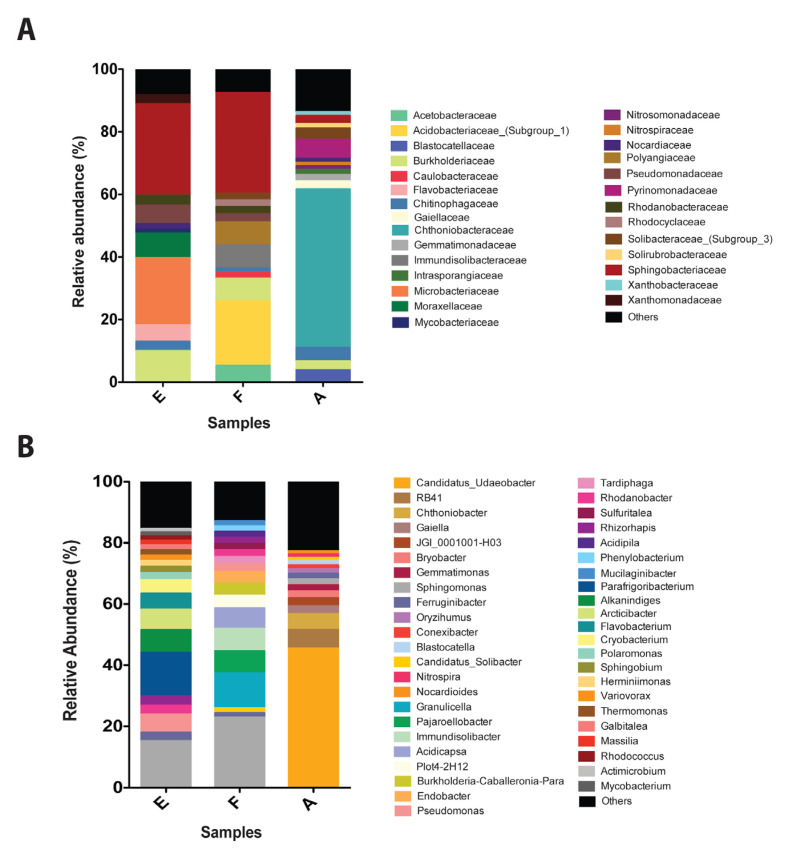
Bacterial communities in Antarctic soil samples exposed to diesel. (**A**) Relative abundance of the most representative families presents in Antarctic soil samples (>1%) non exposed (A, control) and exposed to diesel (E and F). (**B**) Relative abundance (>1%) of the most representative OTUs, classified by genus, present in Antarctic soil samples (>1%) non exposed (A, control) and exposed to diesel (E and F). Abundances below 1% were assigned as others.

The composition of bacterial communities inhabiting Antarctic soils exposed and non-exposed to diesel (control) was characterized by 16S rRNA metagenomic analysis. Important differences were observed in the communities of control and contaminated sites. Control soils present a high abundance of *Chthoniobacteraceae* (50%), *Pyrinomonadaceae* (6.1%), and *Chitinophagaceae* (4.3%) (Figure 3a). The *Chthoniobacteraceae* family is the most predominant in the control sample and has been previously described as a regular component of different soils [59]. On the other hand, samples exposed to diesel were mainly composed of *Sphingobacteriaceae* (29.3% and 32.3%), *Microbacteriaceae* (21.4%), *Burkholderiaceae* (10%) and *Pseudomonadaceae* (6%) in sample E, and *Acidobacteriaceae*_(Subgroup_1) (21%), *Polyangiaceae* (7.3%) and *Burkholderiaceae* (7.3%) in sample F. All these organisms have been previously identified in soils and extreme environments [60,61].

*Candidatus_Udaeobacter* (45.5%) is the most abundant species in control samples, this organism is commonly present in grassland soils [62,63]. Additionally, *Candidatus_Udaeobacter*, *Sphingomonas* (1.9%), and *Conexibacter* (1.2%) have been observed in post-coal mining soils [64]. RB41 (6.1%) and *Blastocatella* (1.2%) have been identified in tundra soils [65]. Other identified species corresponds to *Chthoniobacter* (5.2%), *Gaiella* (2.6%), JGI_0001001-H03 (2.6%), *Bryobacter* (2.3%), *Gemmatimonas* (1.98), *Ferruginibacter* (1.7%), *Oryzihumus* (1.4%), *Nitrospira* (1.1%), and *Nocardioids* (1%) (Figure 3b).

In general, our results revealed that organisms identified in diesel-exposed soils are rich in species associated with contaminated soils. In sample E, OTUs with higher relative abundance are *Parafrigoribacterium* (14%), *Alkanindiges* (7.5%), *Arcticibacter* (6.7%), *Pseudomonas* (6%), *Flavobacterium* (5.2%), *Cryobacterium* (4.5%), *Rhizorhapis* (3%), *Rhodanobacter* (2.9%), *Polaromonas* (2.3%), and *Sphingobium* (2%). On the other hand, in sample F OTUs with the highest relative abundance were *Granulicella* (11.3%), *Pajaroellobacter* (7.3%), *Immundisolibacter* (7.3%), *Acidicapsa* (6.7%), Plot4-2H12 (4.1%), *Burkholderia-Caballeronia-Paraburkholderia* (4%), *Endobacter* (3.8 %), *Pseudomonas* (2.6%), *Tardiphaga* (2.3%), *Rhodanobacter* (2.2%), *Sulfuritalea* (2%), and *Rhizorhapis* (2%). It is interesting to highlight the relative abundance of organisms such as *Sphingomonas* and *Ferruginibacter,* which in samples E and F present a higher relative abundance than control. These organisms are strongly associated with fuel and heavy metals contaminated soils [44,66,67,68,69,70]. Other studies on microbial diversity in diesel polluted soil samples from Antarctica describe higher relative abundances in *Corynebacterineae*, *Flavobacterium*, *Conexibacteraceae,* and *Sphingobacteriales* [71]. All these organisms were identified in sample E and F. Additionally, a variation in the microbial community has been described in Antarctica’s polluted nearshore [42,72]. The presence of bacterial species associated with the degradation of fuels and heavy metals such as *Sphingomonas*, *Ferruginibacter*, *Pseudomonas*, *Rhodanobacter*, and *Sphingobium* on diesel-exposed soils, suggest an effect of co-contamination on bacterial communities.

### 3.3. Biological Activity of Diesel-Exposed Soils Is Less Affected by Cadmium than Control Soils

**Figure 4:**
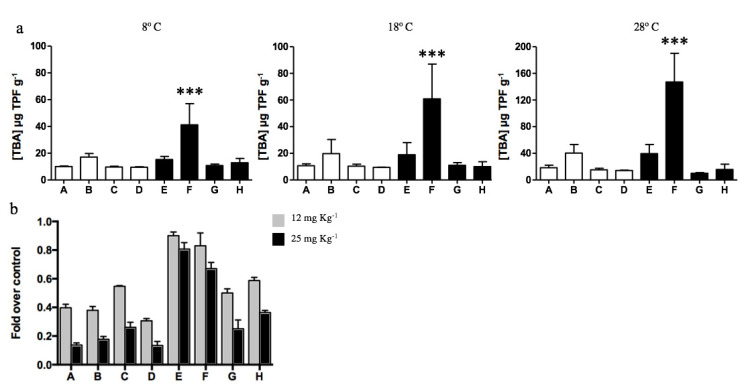
Dehydrogenase activity of soil samples determined at different temperatures. (**a**) Total biological activity (TBA) measured by TPF g^−1^ generated in non-exposed (white bars; A, B, C, and D) and diesel-exposed soils (black bars; E, F, G, and H). The assay was performed at 8, 18, and 28 ℃. *** Represents significance with a *p* < 0.0001. (**b**) Fold decrease on CFUs g^−1^ determined on soils exposed to 12 (gray bars) and 25 mg kg^−1^ of cadmium (black bars) at 28 ℃. CFUs g^−1^ determined in the absence of cadmium correspond to 100%.

The total dehydrogenase activity of soils was determined to evaluate the effect of diesel contamination on biological activity. No significant differences were determined among all samples analyzed, excepting diesel-contaminated sample F that presented the highest dehydrogenase activity. Soil sample F displayed a biological activity 3, 5, and 6.7 times higher than the other 7 samples when evaluated at 8, 18, and 28 °C, respectively. The metabolic activity of microorganisms inhabiting Antarctic soils increased with the temperature, and the highest activities were obtained at 28 °C for all soil samples (Figure 4a). As has been regularly reported during recent years [52,73,74,75], most culturable microorganisms inhabiting Antarctic soils (particularly at King George Island) present optimal growth temperatures near 20–28 ℃. Although psychrotolerant bacterial communities can survive at low temperatures, their metabolic and division rates are highly affected.

Then, the effect of cadmium on the dehydrogenase activity of soils was analyzed at 28 °C in vitro. Cadmium concentrations of 12 and 25 mg kg^−1^ in non-diesel-exposed soils generated a significant decrease in biological activity, on average 60% and 85%, respectively (Figure 4b). Biological activity was less affected in diesel-exposed soils. Interestingly, the biological activities of samples E and F were weakly inhibited by cadmium exposure. In the case of soil E, 10% and 19% reduction in biological activity was measured after exposure to 12 and 25 mg kg^−1^ cadmium, respectively. Meanwhile, sample F displayed a 17% and 31% reduction in dehydrogenase activity after exposure to the same cadmium concentrations.

### 3.4. Diesel-Exposed Soils Present an Increased Number of Cadmium-tolerant and PAH Metabolizing Bacteria

**Figure 5:**
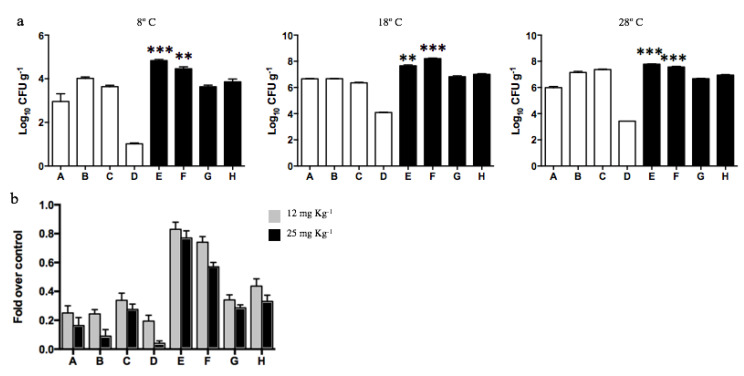
Bacterial counts (CFU g^−1^) determined in Antarctic soil samples. (**a**) Log10 CFU g^−1^ were determined in soils non-exposed to diesel (white bars; A, B, C, and D) and exposed to diesel (black bars; E, F, G, and H). The assay was performed at 8, 18, and 28 °C. ** and *** represents significance with a *p* < 0.001 and *p* < 0.0001, respectively. (**b**) Effect of cadmium over CFUs g^−1^ present on soils was analyzed in vitro at 28 °C in presence of 12 (gray bars) and 25 mg kg^−1^ of cadmium (black bars).

Since dehydrogenase measurements indicated that exposure to diesel improves the metabolic activity of soil F, we decided to determine the total number of cultivable heterotrophic bacteria (CFU g^−1^) present in all soil samples. The analyses were performed at 8, 18, and 28 °C. In agreement with the results of dehydrogenase activity shown in Figure 3a, the CFU g^−1^ values determined in all samples increased at higher incubation temperatures (Figure 5a). This increase was particularly significant for sample F that presented over 3000 times CFUs g^−1^ at 28 ℃. Moreover, samples E and F showed almost 2, 3, and 5 times more CFUs g^−1^ than the other samples at 8, 18, and 28 °C, respectively. The highest numbers of heterotrophic bacteria were determined in those samples exhibiting increased levels of PAHs and metals (E and F), thus supporting the hypothesis that diesel exposure is affecting the microbial communities.

Since the dehydrogenase activity of diesel contaminated soils was scarcely affected by the presence of the heavy metal cadmium, we decided to determine the number of cadmium-tolerant heterotrophs present in each Antarctic soil (Figure 5b). The exposure of soil samples not contaminated with diesel to 12 or 25 mg kg^−1^ cadmium generated, on average, a 75% and 89% decrease in CFU g^−1^, respectively. In contrast, diesel-exposed soils, particularly samples E and F, evidenced an effect of lower magnitude when exposed to cadmium 12 mg kg^−1^ (17% and 26% decrease, respectively) or 25 mg kg^−1^ (29% and 43% decrease, respectively). These results confirm the selection of cadmium-tolerant bacteria in soils exposed to diesel.

Based on obtained results indicating that diesel contamination of Antarctic soils is associated with increasing levels of heavy metals and PAHs, thus favoring the establishment of metal tolerant microorganisms, we decided to determine the number of bacteria capable of metabolizing PAHs present in the soils. To this end, the numbers of cultivable environmental bacteria in diesel-exposed and control samples were determined. After incubation for 5 days at 28 °C in solid R2A media, 111 and 239 isolates were obtained from non-exposed and diesel-exposed samples, respectively. From these isolates, bacterial cells capable of using phenanthrene (a three-ring PAH) as the only carbon source were determined by growing each colony in M9 media supplemented with phenanthrene. Bacterial growth was determined by measuring OD600 and PAH degradation was detected by the generation of a dark color in the culture as reported previously [44,57]. Only 4 PAH-metabolizing isolates were obtained from samples not exposed to diesel. Meanwhile, 49 isolates capable of metabolizing phenanthrene were obtained from diesel-exposed samples. This result confirms that the exposure of Antarctic soils to diesel is selecting for bacteria capable of tolerating and metabolizing PAHs.

**Figure 6:**
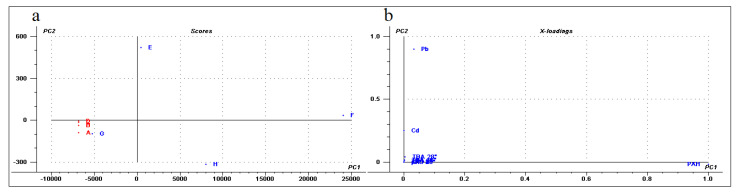
Principal Component Analysis. The analysis was performed with the software “The Unscrambler”. (**a**) The soil samples projected in the first two components (score plot). Each sample was colored according to their condition, red (control) or blue (exposed to diesel). (**b**) The value of the variable coefficient for the first two components (loading plot) using values of heavy metal concentrations, PAH concentration, phenanthrene-degrading isolates and TBA at different temperatures.

Finally, a multivariate general characterization of the samples was performed by principal component analysis (PCA). For this analysis, we also evaluated the phenanthrene-metabolizing bacteria isolated from these Antarctic soils described in previous work [44]. The first two components explain 87% of the variance, the first one is mainly explained by lead concentration and to a lesser extent by bacterial isolates, PAH concentration, and soil dehydrogenase activity. In the case of the second component, these variables and the concentrations of cadmium and chromium are the most relevant (Figure 6). Consequently, control soils and samples G and H were grouped close to the center of the plot (Figure 6). This is associated with a lower contribution of each of the described variables. Meanwhile, samples E and F were distanced from the central group and were associated with higher contents of metals or bacterial isolates, PAHs, and biological activity, thus confirming a direct association between bacterial content/biological activity and PAH/metal concentrations. In general, the PCA analysis revealed the existence of significant differences between diesel-exposed and control samples.

## 4. Discussion

In the present work, a chemical and biological characterization of diesel contaminated Antarctic soils was performed to evaluate the level of contamination and its effect on soil microorganisms.

### 4.1. PAHs and Metal Contamination on Diesel-Exposed Antarctic Soils

Antarctica has been exposed to oil contamination for decades and PAH presence has been reported in diesel-exposed soils from this continent. The obtained results indicate that exposed soils contain concentrations of three-ring PAHs 1000 times higher than control soils reflecting the impact of diesel pollution generated by human activities. High concentrations of PAHs were determined in oil-exposed soil samples of King George Island (between 0.9–30.8 mg kg^−1^). In general, the concentrations of PAHs determined in Antarctic diesel-exposed soils or sediments are in the range of 10–40 mg kg^−1^ and the level of non-exposed samples ranges from 10–100 µg kg^−1^ [2]. At King George Island, PAH concentrations of 28 and 19–42 mg kg^−1^ have been reported in sediments and soils, respectively [76]. Recently, Dauner et al. (2015), reported PAH concentrations of 12.5–210 mg kg^−1^ in sediments of the Shetland Islands [77]. On James Ross Island sediments, PAH concentrations of 1.4–205 and 17.1–34.9 mg kg^−1^ were determined in sediments and soils, respectively [78].

Diesel contaminated samples analyzed in our work presented metal concentrations ranging from 28.2–743, 9.59–81.3, 0.14–1.26 mg kg^−1^ for lead, cadmium, and chromium, respectively. Control samples displayed a lower concentration of cadmium, lead, and chromium when compared to diesel-exposed soils. Interestingly, soil samples with the highest levels of PAHs (samples E and F) also showed the highest levels of heavy metals. Metals concentrations determined in diesel contaminated sites are higher than most metal concentrations previously reported in King George Island, particularly in the case of cadmium and lead [2]. Our results agree with previous publications, particularly in the case of soils not exposed to diesel. However, the levels of cadmium and lead determined in diesel contaminated samples are much higher than those previously reported in Antarctic samples, illustrating the importance of diesel contamination on soil quality [2,15,19,20,21].

Heavy metals and PAH concentrations determined in diesel-exposed soils of King George Island are evidence of high levels of contamination that may affect the different organisms inhabiting this zone, particularly the microorganisms. Concerning the international regulations, the Chilean environmental normative establishes that concentrations of cadmium, chromium, and lead may not exceed 40, 150, and 400 mg kg^−1^, respectively [79]. In general, cadmium, chromium, and lead concentrations below 40, 380, and 530 mg kg^−1^ are permitted in soils of countries such as Holland, Canada, the UK, and Australia [80,81,82,83]. Obtained results indicate that none of the samples exceeds the normative for chromium concentration. However, two soil samples are above lead standards (E and F), and four samples exceed the cadmium concentrations permitted by the regulations of these countries (C, E, F, and H).

### 4.2. Bacterial Communities Inhabiting Diesel-Exposed Soils

The evaluation of quantitative and qualitative parameters of environmental microorganisms provides an effective approach to assess the effects of contamination since these communities are very sensitive to changes and respond rapidly to environmental perturbations. It is known that in microbial communities inhabiting environments chronically exposed to hydrocarbons, a selection for organisms capable of utilizing PAH as carbon and energy source occurs [6,42,72,84].

To determine the effect of diesel contamination on bacterial composition, we evaluated those samples that presented the highest concentration of PAHs and metals (samples E and F). The 16S metagenomic analysis of soils revealed important differences in the composition of bacterial communities inhabiting control and diesel contaminated soils. *Sphingomonas* relative abundance was higher in soil samples exposed to diesel than in controls. This genus has been determined in petroleum-contaminated soils as part of the organisms capable of degrading PAHs [66,67,68]. In general, the species identified in the soil samples exposed to diesel are related to PAHs, diesel, aromatic compounds, and heavy metal contamination. Previous studies have identified a high abundance of phyla’s such as *Proteobacteria*, *Bacteroidetes*, *Actinobacteria*, *Chloroflexi*, and *Acidobacteria* using 16S rRNA sequencing and pyro-sequencing in soils exposed to heavy metals such as mercury, arsenic, lead, and cadmium [69,70,84]. Antarctic soils exposed to diesel E and F were rich in *Proteobacteria*, *Bacteroidetes*, *Actinobacteria*, and *Acidobacteria*. In previous research, we isolated a bacterium of the genus *Sphingobium* from diesel-contaminated Antarctic soils. Interestingly, this *Sphingobium* isolate had a high capacity to degrade PAHs in the presence of heavy metals such as chromium and cadmium [44]. In this regard, our metagenomic analysis revealed an enrichment of organisms with the ability to degrade aromatic compounds: *Sulfuritalea* [85], *Rhodanobacter* [86], and *Massilia* [87]; degrade PAHs: *Sphingobium* [44], *Pseudomonas* [43,84,88], and *Immundisolibacter* [89], and also degrade petroleum: *Phenylobacterium* [90].

In this context, the results presented in our work confirm that diesel exposure has favored the development of microorganisms that can use hydrocarbons as an energy source. Moreover, the data obtained revealed that microorganisms present in diesel-exposed samples are less sensitive to cadmium exposure than microorganisms inhabiting control soils. This result is probably a consequence of a selection pressure generated by the exposure to high concentrations of cadmium present in diesel contaminated soils.

### 4.3. Effect of PAHs and Cadmium on Dehydrogenase Activity, CFUs, and PAHs Degrading Bacteria

It is known that bacteria and fungi can use some diesel components as a carbon source or as electron donors to obtain energy [6,44]. Total soil dehydrogenase activities and CFUs determined in diesel-exposed soils suggest an environmental selection for microorganisms capable of metabolizing hydrocarbons and growth in presence of high concentrations of cadmium.

The effect of metals on microorganisms and their processes depends on the available or free concentration of the metal and not on the total concentration. In this context, the presence of microorganisms capable of tolerating cadmium and metabolizing PAHs suggests that the metal is directly interacting with the microbial community. This is highly relevant since it is known that PAH biodegradation is particularly sensitive to cadmium and small concentrations of the metal can strongly affect degradation rates [28].

Different studies on hydrocarbon contaminated soils in Antarctica have revealed that although the presence of hydrocarbons strongly influences the diversity of microbial communities, other factors such as pH, soil structure, and the C/N content are also relevant [36,39]. In this context, it is likely that other variables, apart from the concentrations of PAHs and heavy metals, also contribute to modulating the microbial communities of diesel contaminated soils of King George Island.

The PCA analysis performed in this study showed significant differences between the control and exposed samples. The PC2 was correlated predominantly with the lead concentration, mainly due to the chemical composition of sample E. This statistically separated the samples E and H from all the others, either by a positive correlation for sample E with the lead concentration or by a negative correlation with lead for sample H. Finally, the PCA also suggested a correlation for the PAH level driven by sample F.

Understanding the effect of co-contamination on hydrocarbon biodegradation or regulating the dynamics and properties of microbial communities would strongly contribute to the development of specific bioremediation strategies to achieve the decontamination of this unique environment affected by diesel spills. Based on the results reported in the present work, we are currently working on studying the effects of different metals (Cd, Pb, and Cr) on PAH biodegradation by Antarctic bacteria inhabiting soils of scientific bases located in different zones of Antarctica.

The obtained results are promising for future work on the Antarctic bioremediation of diesel-exposed soils, particularly the existence in these soils of bacteria that can tolerate heavy metals. This point is highly relevant since most microorganisms used in bioremediation processes have lower activity in the presence of co-contaminants such as metals [91]. Thus, potential bioremediation processes in Antarctic soils could be improved by using bacterial pure cultures or consortiums capable of degrading PAHs in the presence of co-contaminants such as cadmium or lead.

## Figures and Tables

**Table 1:** Location and characteristics of Antarctic soil samples.

Sample	Exposure to Diesel	Latitude	Longitude	Temperature
A	Non-exposed	62°25 S	59°46 W	1 °C
B	Non-exposed	62°26 S	59°23 W	3 °C
C	Non-exposed	62°38 S	60°36 W	3 °C
D	Non-exposed	62°58 S	60°34 W	3 °C
E	Exposed	62°19 S	57°53 W	0 °C
F	Exposed	62°19 S	57°55 W	0 °C
G	Exposed	62°11 S	58°58 W	5 °C
H	Exposed	62°13 S	58°57 W	9 °C

## Supplementary Materials

The following are available online at https://www.mdpi.com/2076-2607/8/11/1749/s1, Figure S1. Localization of analyzed soil samples. Eight soil samples taken from South Shetland Island were analyzed in this study, samples A–D correspond to control with no diesel contamination. Samples E-H were exposed to diesel. Figure S2. pHs of selected Antarctic soils. (A) The pH of control (white bars) and diesel exposed samples (black bars) was determined (three soil samples of each site were evaluated). (B) The average pH of control and exposed samples was determined. Tukey statistical analysis of multiple comparisons determined significant differences between both sets of samples, *p*-value < 0.05. Table S1. Summary table of chemical quantification on Antarctic soil samples. The content (mg Kg^−1^) of cadmium (Cd), chromium (Cr), lead (Pb) and phenanthrene were measured.
