# Supplementary material for: Effect of Co-contamination by PAHs and Heavy Metals on Bacterial Communities of Diesel Contaminated Soils of South Shetland Islands, Antarctica
Source: Microorganisms. 2020 Nov 7;8(11):1749. doi: 10.3390/microorganisms8111749 (PMC7695015; doi:10.3390/microorganisms8111749)
Supplement: Supplementary file 1 [file microorganisms-08-01749-s001.pdf]

**Supporting Information**

**Figure S1.** Localization of analyzed soil samples. Eight soil samples taken from South Shetland Island were analyzed in this study, samples A-D correspond to control with no diesel contamination. Samples E-H were exposed to diesel.

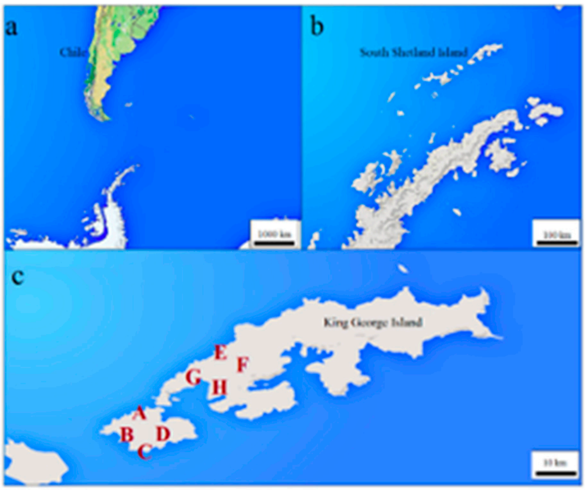


**Figure S2.** pHs of selected Antarctic soils. (A) The pH of control (white bars) and diesel exposed samples (black bars) was determined (three soil samples of each site were evaluated). (B) The average pH of control and exposed samples was determined. Tukey statistical analysis of multiple comparisons determined significant differences between both sets of samples, p-value < 0,05.

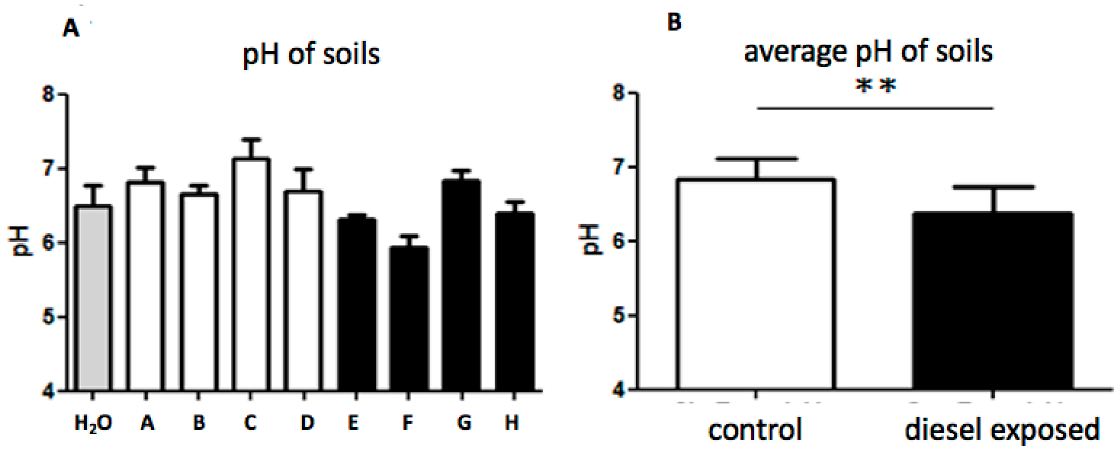

**Table S1.** Summary table of chemical quantification on Antarctic soil samples. The content (mg $Kg^{-1}$) of cadmium (Cd), chromium (Cr), lead (Pb), and phenanthrene were measured.

| [mg $Kg^{-1}$] | Sample | | | | | | | |
|---|---|---|---|---|---|---|---|---|
| | Nelson Island | | | | Fildes Peninsula | | | |
| | A | B | C | D | E | F | G | H |
| Cd | 9.79 | 7.51 | 18.4 | 3.35 | 81.3 | 28.9 | 9.59 | 21.9 |
| Cr | 0.31 | 0.97 | 0.24 | 0.32 | 1.26 | 0.85 | 0.36 | 0.14 |
| Pb | 6.24 | 57.9 | 80.2 | 88.6 | 743 | 678 | 28.2 | 44 |
| Phenanthrene | <0.01 | 0.04 | <0.01 | <0.01 | 7.3 | 30.8 | 0.9 | 14.9 |
